# Characterization of *WRKY* Gene Family in Whole-Genome and Exploration of Flowering Improvement Genes in *Chrysanthemum lavandulifolium*

**DOI:** 10.3389/fpls.2022.861193

**Published:** 2022-04-26

**Authors:** Muhammad Ayoub Khan, Kang Dongru, Wu Yifei, Wang Ying, Ai Penghui, Wang Zicheng

**Affiliations:** State Key Laboratory of Crop Stress Adaptation and Improvement, Plant Germplasm Resources and Genetic Laboratory, Kaifeng Key Laboratory of Chrysanthemum Biology, School of Life Sciences, Henan University, Kaifeng, China

**Keywords:** *WRKY* transcription factors, ornamental, motifs, *cis*-acting elements, CpG islands, methylation, flowering traits

## Abstract

Chrysanthemum is a well-known ornamental plant with numerous uses. *WRKY* is a large family of transcription factors known for a variety of functions ranging from stress resistance to plant growth and development. Due to the limited research on the *WRKY* family in chrysanthemums, we examined them for the first time in *Chrysanthemum lavandulifolium*. A total of 138 *ClWRKY* genes were identified, which were classified into three groups. Group III in *C. lavandulifolium* contains 53 members, which is larger than group III of *Arabidopsis*. The number of introns varied from one to nine in the *ClWRKY* gene family. The “WRKYGQK” motif is conserved in 118 members, while other members showed slight variations. AuR and GRE responsive *cis*-acting elements were located in the promoter region of *WRKY* members, which are important for plant development and flowering induction. In addition, the W box was present in most genes; the recognition site for the *WRKY* gene may play a role in autoregulation and cross-regulation. The expression of the most variable 19 genes in terms of different parameters was observed at different stages. Among them, 10 genes were selected due to the presence of CpG islands, while nine genes were selected based on their close association with important *Arabidopsis* genes related to floral traits. *ClWRKY36* and *ClWRKY45* exhibit differential expression at flowering stages in the capitulum, while methylation is detected in three genes, including *ClWRKY31, ClWRKY100*, and *ClWRKY129.* Our results provide a basis for further exploration of *WRKY* members to find their functions in plant growth and development, especially in flowering traits.

## Introduction

*Chrysanthemum lavandulifolium* is one of the original species of *Chrysanthemum* **×** *morifolium* and uses as a model plant. It is a major ornamental plant, originated in China, an important cut flower with diversified petal color and shape having a variety of flower structures, which makes it a highly valuable ornamental plant in the floral industry worldwide (Nguyen and Lim, 2019). Chrysanthemum is among the four most important ornamental plants in the world used for beautification and aesthetic value (Shinoyama et al., 2012). The flower of chrysanthemum is also known for its health benefits, and it contains important antioxidants and phenolic compounds (Lin and Harnly, 2010; Yue et al., 2018).

Due to a simple diploid (2*n* = 2*x* = 18) genetic background, *C. lavandulifolium* dominates over *C. morifolium* (Huang et al., 2012; Dong-ru et al., 2019). The wild ancestors having desired qualities against biotic and abiotic cues can be used for the genetic improvement of cultivated varieties. Identification and incorporation of such genes into commercial varieties reduces pesticide use, improves plant health, and induces photoperiod-controlling flowering mechanisms (Wu et al., 2010; Fu et al., 2013; Yang et al., 2017).

Genetic architectural study is one of the major research directions in any plant. By narrowing the path further, transcription activation and silencing is the major field of concern. Many transcription factors (TFs) have been explored to switch on and off against certain environmental conditions. *WRKY* TF is one of the major families first identified in plants in 1994 by the name of DNA-binding protein in sweet potato, which is labeled as *SWEET POTATO FACTOR1 (SPF1)* (Ishiguro and Nakamura, 1994). *WRKY* TFs play important roles in resistance against biotic (fungal or bacterial pathogen) and abiotic stresses, growth, and development. Several *WRKY* TFs have been proven to provide resistance against biotic stresses exerted by fungal or bacterial pathogens by influencing other associated genes (Turck et al., 2004; Duan et al., 2007; Zhou et al., 2008, 2011; Li et al., 2009, 2011; Chen et al., 2010; Scarpeci et al., 2013; Phukan et al., 2016; Yokotani et al., 2018; Warmerdam et al., 2020). The name *WRKY* has been given to this family for their distinguishing characteristic, the *WRKY* domain, which is composed of highly conserved 60 amino acids and a zinc finger motif. *WRKY* TFs are classified into three groups based on the number of (WRKYGQK) domain, and zinc-finger motif. Group I has two domains, and groups II and III have one domain. Groups I and II have C2H2 type zinc finger motifs while group III has a C_2_HC zinc finger motif. Members of group II were further classified into subgroups (Rushton et al., 2010).

*WRKY* TFs are extensively studied in *Arabidopsis* and most other plants. *WRKY*7 and *OsWRKY11* expression resulted in blooming delay in *Arabidopsis* and rice (Cai et al., 2014; Chen et al., 2019), *WRKY13* has an adverse effect on flowering time, and *WRKY*12, *GsWRKY20* and *CsWRKY50* promote flowering, possibly through the influence of gibberellic acid under short-day conditions in *Arabidopsis* (Luo et al., 2013; Kumar et al., 2016; Li et al., 2016), *GhWRKY22* regulating pollen development (Wang et al., 2019; Ramos et al., 2021). While different *WRKY* family TFs play an important role in chrysanthemum, they showed resistance to salt stress (Liu et al., 2013, 2014; Li et al., 2015b; Jaffar et al., 2016; Liang et al., 2017; Wang K. et al., 2017; He et al., 2018; Zhang et al., 2020), and *CmWRKY1* and *CmWRKY15* transgenic lines in *C. morifolium* interact with *SA* and *ABA* and altered plant development (Fan et al., 2015, 2016; Bi et al., 2021). *CmWRKY48* showed resistance against aphid infestation (Li et al., 2015a). They also play a regulatory role in the flowering stages. The complex network of *VQ20*, *WRKY2*, and *WRKY34* interaction influences the plant gametogenesis and plays a crucial part in the development of pollen and pollen tube growth, modulating flowering (Lei et al., 2017; Ma et al., 2020).

Flowering time is a complex character regulated by a network of different external and internal signals. Different pathways have been identified, including photoperiod pathway, vernalization pathway, gibberellin-regulated pathways, and autonomous floral initiation pathway (Soares et al., 2020). Therefore, flowering induction studies are more crucial in ornamental plants, especially in chrysanthemum, where all the ornamental characteristics are associated with flowering, i.e., flower color, shape, and size. Chrysanthemum is a short-day plant and needs a specific photoperiod to induce flowering, which makes it relatively expensive as well as limits the annual production of chrysanthemum flowers. We explored the *WRKY* gene family in *C. lavandulifolium* for the first time, prior, the *WRKY* gene family has only been studied in the *C. morifolium* (Song A. et al., 2014). The size of the *WRKY* family in *C. morifolium* comprised 15 members, which were named from *CmWRKY1* to *CmWRKY15* (GenBank: KC615355–KC615369). As *WRKY* is a large family, influencing floral characteristics, we need to explore them deeply. Our present study was a part of such investigation in which we tried to explore the *WRKY* gene family in *C. lavandulifolium* comprised structural gene analysis, protein motifs, phylogenetic relatedness of *C. lavandulifolium* and *Arabidopsis thaliana*, *cis*-acting elements, recognition of CpG rich regions, the methylation status of the genes, and expression of 19 important genes in different tissues at different stages to check their role in flowering-related traits. This investigation will lead us to identify variation in transcription regions in the *WRKY* family for future chrysanthemum research.

## Materials and Methods

### Plant Material and Treatment

The plant materials of *C. lavandulifolium* were obtained from Beijing Forestry University. The plants’ G1 lines were grown under controlled environmental conditions in the growth chamber. All the plants used in this research came from the G1 line. The plants were propagated by cuttings and grown in pots. The plants were kept under long-day conditions (14 h L/10 h D) light/dark at a 25°C temperature. The photoperiod conditions were changed to short-day conditions (12 h L/12 h D) when the plants attained maturity (more than 14 leaves). The temperature was kept constant. The plants were watered every 7 days. Stem, root, leaf, buds, and capitulum samples for quantitative real-time polymerase chain reaction (qRT-PCR) were taken at different growth stages, including young, mature, and flowering. The plants were considered at a young stage when they attained eight leaves. The plants were called into the adult stage when the number of leaves was 14, and they were transferred to short-day conditions. The flower develops in short conditions, and samples were taken at different stages of flower development (Supplementary Figure 1). The little buds (1–3 mm in diameter) appeared after 7 days in short-day conditions and were taken for RNA extraction. The median buds (5–8 mm in diameter) were taken after a few days before flower opening. The first flower samples were taken when the flowers had just opened.

### Characterization of *WRKY* Gene Family in *Chrysanthemum lavandulifolium*

The *C. lavandulifolium* genome has been sequenced (Wen et al., 2022). All the protein and genomic sequences of *ClWRKY* members were obtained from PRJNA681093, National Center for Biotechnology Information (NCBI). The genomic information of *C. lavandulifolium* was completed by Henan University and Beijing Forestry University, and related papers have been published (Wen et al., 2022). The redundant sequences, which lack the *WRKY* domain, were removed after the sequence analysis using manual inspection in the Mega X software. A total of 138 definite *ClWRKY* sequences were taken for further study. The *ClWRKY* members were named from *ClWRKY1* to *ClWRKY138*, according to the source provider (Supplementary Table 3).

### Classification of *ClWRKY* Proteins

Multiple protein sequence alignments of *C. lavandulifolium* and *A. thaliana WRKY* were made using ClustalX and MEGA-X version 10.2.6 (Molecular Evolutionary Genetics Analysis) (Kumar et al., 2018). The phylogenetic tree based on amino acid (aa) sequences of *ClWRKY* and *AtWRKY* conserved *WRKY* domains was constructed following the neighbor-joining method with 1,000 bootstraps to determine the close association of both gene families.

### Gene Structure and Protein Motif Analysis

The gene structure (intron-exon) of *ClWRKY* members, full-length gene, and coding (CDS) sequences were analyzed in the online database, Gene Structure Display Server (GSDS)^1^ as described (Hu et al., 2015). Analysis of the conserved protein motifs was performed using the NCBI CDD batch^2^ and the TBtools software (Chen et al., 2020).

### *Cis*-Acting Elements in the Promoter Region of *ClWRKY* Members

The promoter sequences of all 138 *ClWRKY* members were submitted to the PlantCARE software to analyze their promoter region for *cis*-acting elements (Lescot et al., 2002). For this purpose, a 2,000 bp sequence upstream of the gene was taken to identify *cis*-acting elements. The MEME suite software was used to identify *de novo* motifs and known enriched motifs (Bailey et al., 2015). The *de novo* motif analysis was performed to identify significant patterns (Bailey and Elkan, 1994).^3^ The known enriched motifs were identified *via* simple enrichment analysis using the MEME software (Bailey and Grant, 2021).^4^ Tomtom motif comparison tool was used to quantify the similarity between motifs (Gupta et al., 2007).

### CpG Island Analysis of the Promoter Region of *ClWRKY* Gene Family

For CpG analysis, promoter regions from the *WRKY* gene family were sequenced. For the exploration of CpG islands, the “Methyl Primer Express version 1.0” software was used (Methyl Primer Express Software v1.0 Quick Reference Card).^5^ All the sequences were submitted to the software one by one to identify CpG islands or rich regions. The minimum length of the island was kept at 200 bp, while the maximum length was 2,000 bp. The criterion of “C + G/Total bases” was 50%. The CpG observed/CpG expected ratio was 0.6.

### Genomic DNA Extraction and Bisulfite Treatment

The total DNA was extracted from different tissues of *C. lavandulifolium*. The tissues included root, stem, leaf, and capitulum at three different growth stages, i.e., young stage, adult stage, and flowering stage. Total genomic DNA was extracted using the “Plant Genomic DNA Kit” (TOLOBIO), according to the manufacturer’s instructions. DNA was quantified using Thermo Scientific NanoDrop™ 2000/2000c Spectrophotometers, and the quality of DNA was checked by 1.5% agarose gel electrophoresis.

Total genomic DNA from each sample was treated with sodium bisulfites using the “DNA Bisulfite Conversion Kit” (TIANGEN) according to the manufacturer’s instructions. The conversion efficiency and quality were measured using Thermo Scientific NanoDrop™ 2000/2000c Spectrophotometers.

### Methylation-Specific Polymerase Chain Reaction

Ten genes were selected on the basis of CpG analysis. Primers for all ten genes were designed using the “Methyl primer express version 1.0” software. For this purpose, two types of primers (methylated and unmethylated) were designed to check the possible methylated sites. The primer length ranged from 18 to 22 bp. The PCR amplicon length was between 100 and 175 bp. Methylation-Specific Polymerase Chain Reaction (MSP) was conducted to analyze the CpG island among the ten selected genes after treatment with bisulfite for methylation status. The MSP conditions were as follows: pre-denaturation for 3 min at 95°C, followed by 34 cycles of the 30s at 95°C and 30 s at 55°C, 1 min at 72°C, and a final extension for 5 min at 72°C. PCR products were resolved onto 1.5% agarose gel and observed under UV transilluminator.

### Quantitative Real-Time Polymerase Chain Reaction

Nineteen (Zhou et al., 2011) variable genes on the basis of CpG island and phylogenetic analysis were selected for gene expression patterns. Primers for all the 19 *ClWRKY* genes were designed using the “Primer Premier 5” software. RNA was extracted from different plant tissues, i.e., root, stem, and leaves at young, adult, and flowering stages, while the capitulum samples, which comprise little buds, median buds, and first flower (right at the time of flower opening), were taken at flowering stages. The RNA was extracted using the RNAprep Pure Plant Plus Kit (spin column) according to the manufacturer’s instructions. The cDNA was synthesized using the “StarScript II First-strand cDNA Synthesis Mix with gDNA Remover.” The expression of all the selected genes was observed in qRT-PCR. The qRT-PCR was carried out on light cycle “Quantageneq225 Fluorescent Quantitative PCR,” using “2 **×** RealStar Green Fast Mixture.” The conditions of PCR were as follows: denaturation at 95°C for 2 min, followed by 40 cycles of denaturation at 95°C for 15 s, annealing at 60°C for 20 s, and extension at 65°C for 5 s. The actin gene of the chrysanthemum was used as an internal control. The data from qRT-PCR for relative expression were analyzed using the 2^–Δ Δ*Ct*^ method proposed by Livak and Schmittgen (2001).

## Results

### Identification and Characterization of *WRKY* Gene Family in *Chrysanthemum lavandulifolium*

**FIGURE 1:**
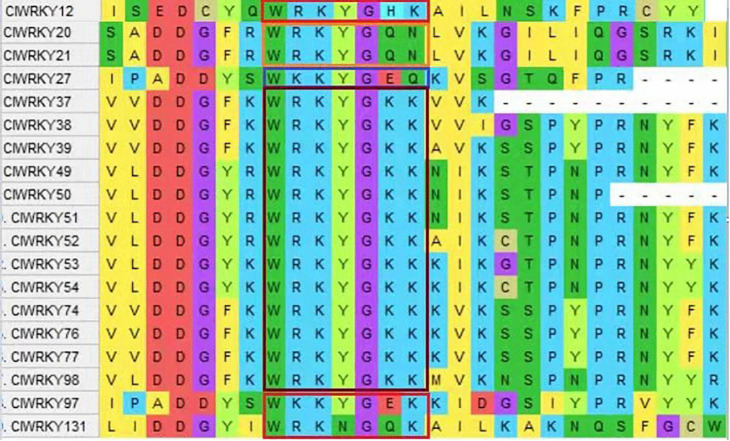
A screenshot of the MEGA X version 10.2.6, showing variation in the conserved domain (WRKYGQK) of *ClWRKY* proteins, and the variation of the conserved domain is encircled in different colors.

*WRKY* genes possess a conserved “WRKYGQK” domain. The “WRKYGQK” heptapeptide is the most defining characteristic of *WRKY* TFs. The *C. lavandulifolium WRKY* sequences were obtained from PRJNA681093, NCBI. A total of 153 *WRKY* gene members have been selected; among them, 138 were exhibited for sequence analysis, and 15 were considered defective. The *WRKY* members are named from *ClWRKY*1 to *ClWRKY*138 according to their location on the chromosome. Protein motif analysis showed homology in most of the conserved regions. Among 138 *ClWRKY* members, 85.51% of members have homology in the “WRKYGQK” core domain, and 19 members have two conserved *WRKY* domains. However, 14.5% of *WRKY* members showed variation in the conserved heptapeptide domain and had substitutions in the core domain. Out of 14.5% variants, 13 members (*ClWRKY*37, 38, 39, 49, 50, 51, 52, 53, 54, 74, 76, 77, and 98) have “WRKYGKK,” substituting glutamine as a lysine aa. *ClWRKY*12 replaced histidine instead of lysine, two genes, *ClWRKY*20 and *ClWRKY21*, shared sixth and seventh as glutamine and asparagine substitutions. High diversity has been found in three genes, i.e., (*ClWRKY*27) “WKKYGEQK,” (*ClWRKY*97) “WKKYGEKK,” and (*ClWRKY*131) “WRKNGQN” *WRKY* domains, respectively (Figure 1).

For the determination of important properties such as aa length, molecular weight, and isoelectric point, we thoroughly analyzed the *WRKY* gene. The average length of *WRKY* proteins was 313 aa, while 74 aa was the minimum and 697 aa was the maximum length. The *WRKY* members with the least and highest lengths were *ClWRKY58* and *ClWRKY36*, respectively. The molecular weight ranged from 0.02 kDa (*ClWRKY55*) to 76.26 kDa (*ClWRKY36*). The pIs (isoelectric point) ranged from 4.73 (*ClWRKY75*) to 10.63 (*ClWRKY97*). Among 138 *ClWRKY* members, 82 were acidic (having less than 7 pI), while 56 were basic (having more than 7 pI). More details of *ClWRKY* features are available in Supplementary Table 1.

### Phylogenetic Analysis and Classification of *ClWRKY* Proteins

**FIGURE 2:**
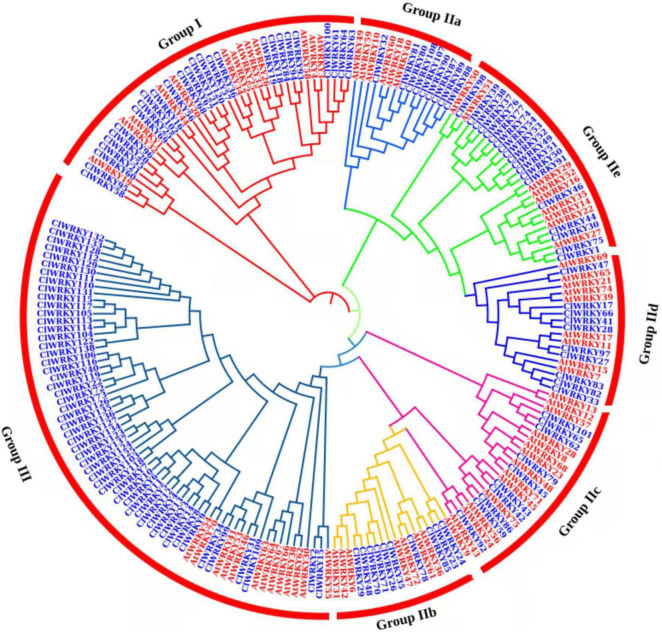
A phylogenetic tree of 138 *ClWRKY* proteins of *Chrysanthemum lavandulifolium* (blue) and 74 *AtWRKY* proteins of *Arabidopsis thaliana* (red) on the basis of amino acid sequences. The tree was constructed in MEGA X. The domains were clustered into three groups, namely, group I, group II, and group III. The group II was divided into five subgroups (a–e).

A phylogenetic tree was constructed between *C. lavandulifolium* and *A. thaliana* to investigate the relatedness of different *WRKY* genes and to understand the classification of different groups among *WRKY* members. *Arabidopsis* is a model plant for genetic study and has been explored for the function of *WRKY* genes. The MEGA-X software was used for agglomerative construction following the neighbor-joining method to find the correlation between *WRKY* members of *C. lavandulifolium* and *A. thaliana*. The *WRKY* members are divided into three groups or seven subgroups. Group I has 27 members, and group II has 58 members with 9 members in II a, 10 members in II b, 10 members in II c, 10 members in II d, and 19 members in II e. Group III has 53 members and was found to be the largest group in this classification (Figure 2). The majority of the *ClWRKY* members clustered in the corresponding groups. Some of the *WRKY* members (*ClWRKY20*, *21, 55, 56*, and *58*) had one *WRKY* domain, but still, clustered in group I; by the rules, group I members should have two domains. These *WRKY* members may evolve from two *WRKY* domain genes and lose one domain in the process of evolution. Previously, such exceptional cases of *WRKY* members with one *WRKY* domain clustering in group I have been reported (Bi et al., 2016).

Some of the *ClWRKY* members clustered with important *Arabidopsis WRKY* genes that helped us identify the genes that are associated with flowering. *ClWRKY36* is clustered with *Arabidopsis AtWRKY2* and *AtWRKY34* (Figure 2), both of which play an important role in pollen development through interaction with the *VQ20* protein (Lei et al., 2017). Similarly, *ClWRKY45* clustered with *AtWRKY71* (Figure 2), which play a key role in the regulation of flowering, directly and indirectly, by influencing other flowering-associated genes (Yu et al., 2016, 2018).

### Gene Structure of *WRKY* Members and Protein Motif Analysis

**FIGURE 3:**
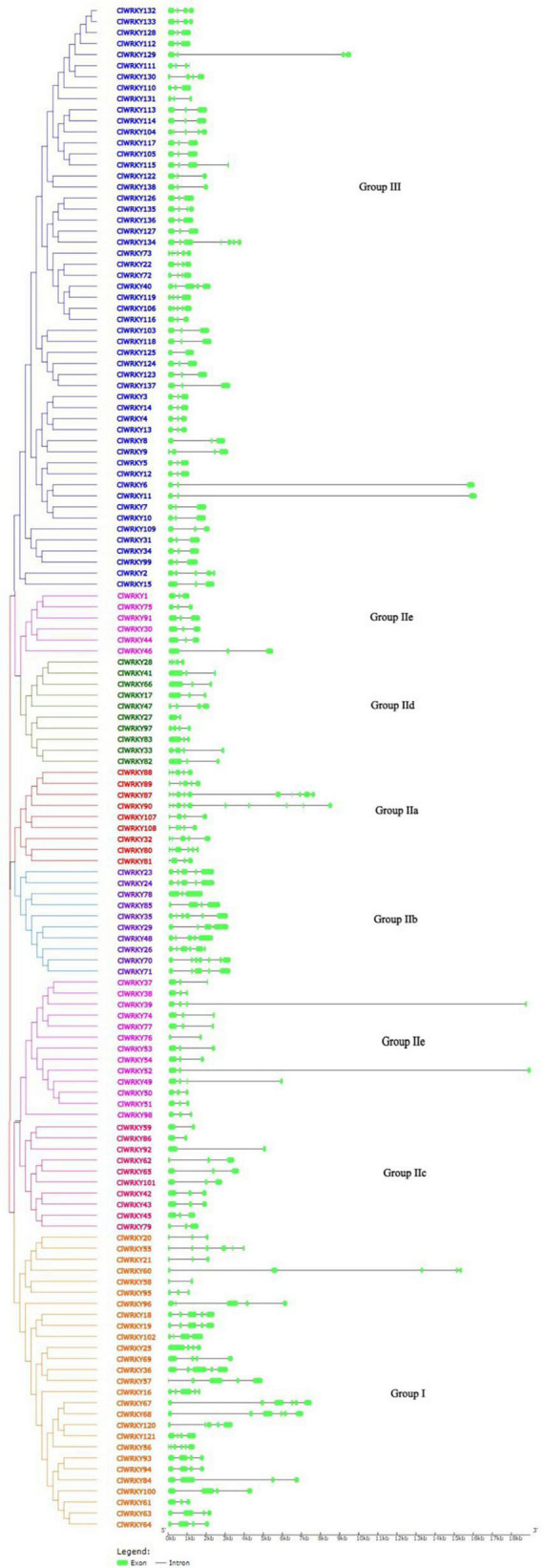
The intron-exon structure of 138 *ClWRKY* members according to the phylogenetic relationship. A phylogenetic tree was constructed with the *ClWRKY* protein sequences using MEGA X. The Gene Structure Display Server (GSDS) software was used to determine the intron-exon structure. Green bars represent exons in the gene. Different colors of *WRKY* members represent different groups in the *ClWRKY* family.

**FIGURE 4:**
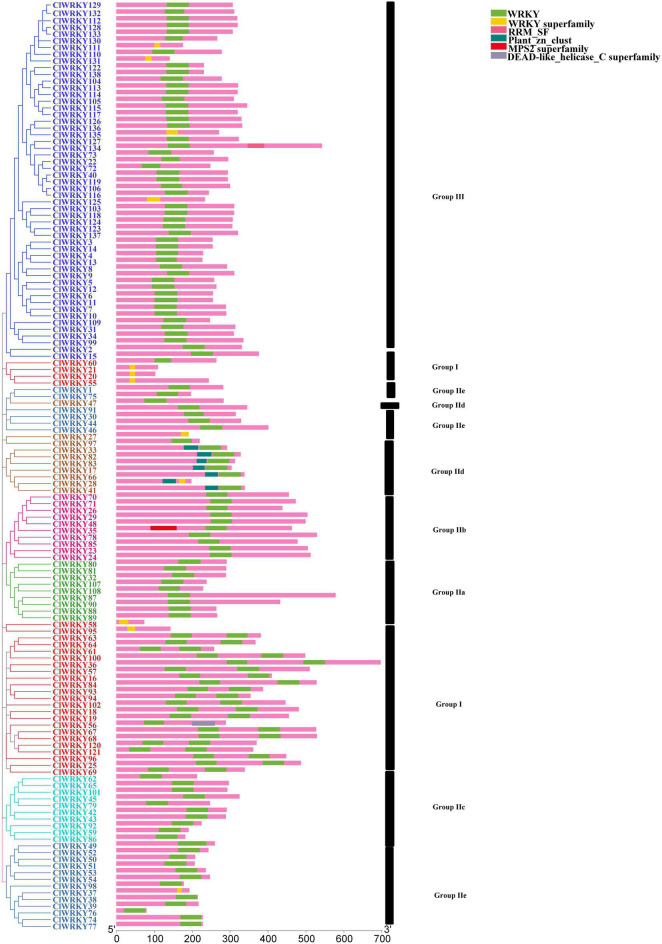
Schematic diagram of conserved motifs in *ClWRKY* proteins according to the phylogenetic relationship. Different color *ClWRKY* members indicate different groups. The conserved motifs were predicted using the TBtools software.

For the determination of intron-exon structure similarity, Gene Structure Display Server (GSDS)^6^ database has been used. Variation among *ClWRKY* members was observed in terms of a number of introns. The minimal number of introns in a gene was one, which occurred in seven members including *ClWRKY27, ClWRKY58, ClWRKY59, ClWRKY76, ClWRKY86, ClWRKY92*, and *ClWRKY125* while *ClWRKY90* had 9 introns, which is the maximal in the whole family. The overall average number of introns in the *ClWRKY* family was three (Figure 3). The number of introns in group I ranged from 1 to 5, group II a varied from 3 to 9, group II b from 2 to 6, group II c from 1 to 2, group II d from 1 to 4, and group II e from 1 to 4. Group III varied from 1 to 6 (Supplementary Table 2). The minimal number of exons was two, which appeared in *ClWRKY27, ClWRKY58, ClWRKY59, ClWRKY76, ClWRKY86, ClWRKY92*, and *ClWRKY125*, while the maximal number of exons was 10, which occurred in *ClWRKY87* and *ClWRKY90* (Supplementary Table 2). The overall average number of exons in the *ClWRKY* family was four (Figure 3). The exon numbers varied within the groups; group I ranged from 2 to 6, group IIa from 4 to 10, group IIb from 3 to 7, group IIc from 2 to 3, group IId from 2 to 5, group IIe from 2 to 4, and group III from 2 to 7, respectively. The average number of exons within the groups is as follows: group I has 4, group IIa has 6, group IIb has 5, group IIc has 3, group IId has 3, group IIe has 3, and group III has 3, respectively (Supplementary Table 2). Protein motif analyses were performed on the *ClWRKY* gene family. The first two motifs represented by green and yellow colors are *WRKY* protein motifs (Figure 4). At least one or two *WRKY* protein motifs have been identified among all the gene sequences. This suggests that *WRKY* is a conserved domain.

### CpG Island Analysis of the Promoter Region of *ClWRKY* Gene Family

CpG island is the region of DNA that is rich in CpG sites and is often located near the promoters of genes. Ten members were found to have CpG islands; among them, seven members contained CpG in their promoter region, two in the cross-promoter and gene region, and one member was found to have it in the gene body. All the ten members had one CpG island. *ClWRKY115* had the shortest island with 438 bp, while *ClWRKY83* had the longest island with a length of 1,065 bp (Table 1). The genes with the CG-rich content are the obvious means of DNA methylation and a prominent source of epigenetic improvement.

**TABLE 1:** Details of each of the ten genes with a CpG island.

NO	*WRKY* Gene	Island #	Start	End	Length	Region
1	*ClWRKY9*	1	–1239	–593	647	Promoter
2	*ClWRKY31*	1	–1049	–251	799	Promoter
3	*ClWRKY33*	1	–4	566	571	Promoter + Gene Body
4	*ClWRKY41*	1	–1296	–688	609	Promoter
5	*ClWRKY83*	1	–2000	–935	1,065	Promoter
6	*ClWRKY97*	1	–80	471	552	Promoter + Gene Body
7	*ClWRKY100*	1	–2000	–1429	571	Promoter
8	*ClWRKY115*	1	–2000	–1562	438	Promoter
9	*ClWRKY129*	1	6494	6950	457	Gene Body
10	*ClWRKY130*	1	–878	–251	628	Promoter

*The 138 ClWRKY genes were analyzed for CG-rich regions using the “Methyl Primer Express version 1.0” software.*

### Identification of DNA Methylation

**FIGURE 5:**
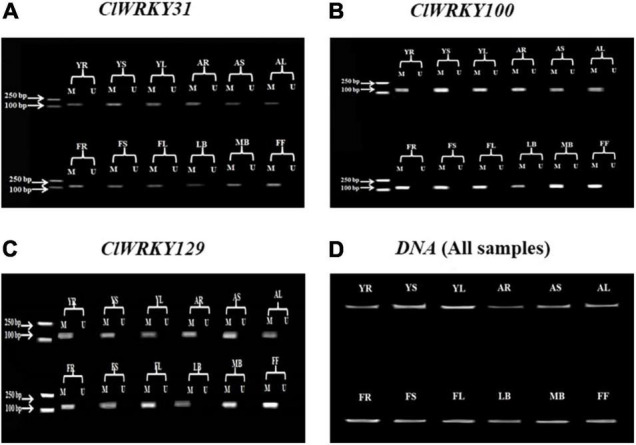
*ClWRKY31, ClWRKY100*, and *ClWRKY129* Methylation-Specific Polymerase Chain Reaction (MSP) results **(A–C)**. An MSP was conducted to determine the methylation status of selected genes. The PCR product length of the genes was as follows: *ClWRKY3*1 = 156 bp, *ClWRKY100* = 135 bp, and *ClWRKY129* = 131 bp. “M” represents a methylated PCR product, and “U” represents an unmethylated PCR product. **(D)** The genomic DNA results for all samples. These results were verified through gel (1.5%) electrophoresis. The abbreviations of different growth stages and tissues are as follows: YR, young stage root; YS, young stage stem; YL, young stage leaf; AR, adult stage root; AS, adult stage stem; AL, adult stage leaf; FR, flowering stage root; FS, flowering stage stem; FL, flowering stage leaf; LB, little buds; MB, median buds; FF, first flower at just opening time.

Total genomic DNA of *C. lavandulifolium* was extracted from the root, stem, leaf, little buds, median buds, and first flowers (flowers at just opening time). These tissues were taken at different growth stages of the plant, which include the young stage, adult stage, and flowering stage. The methylation and unmethylation primers were designed for the ten different genes using the “Methyl Primer Express version 1.0” software. These genes were selected due to the presence of CpG islands. The MSP was carried out for the following ten genes: *ClWRKY9, ClWRKY31, ClWRKY33, ClWRKY41, ClWRKY83, ClWRKY97, ClWRKY100, ClWRKY115, ClWRKY129*, and *ClWRKY130*. The methylation status detected by MSP was analyzed through gel electrophoresis. The MSP results showed the presence of methylation in *ClWRKY31, ClWRKY100*, and *ClWRKY129* at different stages in different tissues. These genes showed significant methylation status in all tissue samples at all growth stages of the plant (Figures 5A–C). However, the methylation status detection through bisulfite sequencing can give us more clear results compared with the MSP method.

### *Cis*-Acting Elements in the Promoter Region of *ClWRKY*

The *cis*-acting elements in the promoter region are really important for gene expression (Rombauts et al., 1999). The 2,000 bp sequence upstream of the genes was submitted to the PlantCARE software and searched for *cis*-acting elements. A range of *cis*-acting elements was identified in the promoter region of the Cl*WRKY* family, which were highly diverse.

A total of 110 *cis*-acting elements were found in the promoter region of *ClWRKY* members. Most of these elements have a diverse role in the plant life cycle. According to our objectives, we screened out the elements that can play a role in plant growth and development and particularly in flowering induction. Therefore, we selected 15 *cis*-acting elements on the basis of their possible role in flowering-related traits and the overall development of the plant. The detailed features of the 15 *cis*-acting elements are provided in Supplementary Table 4.

**FIGURE 6:**
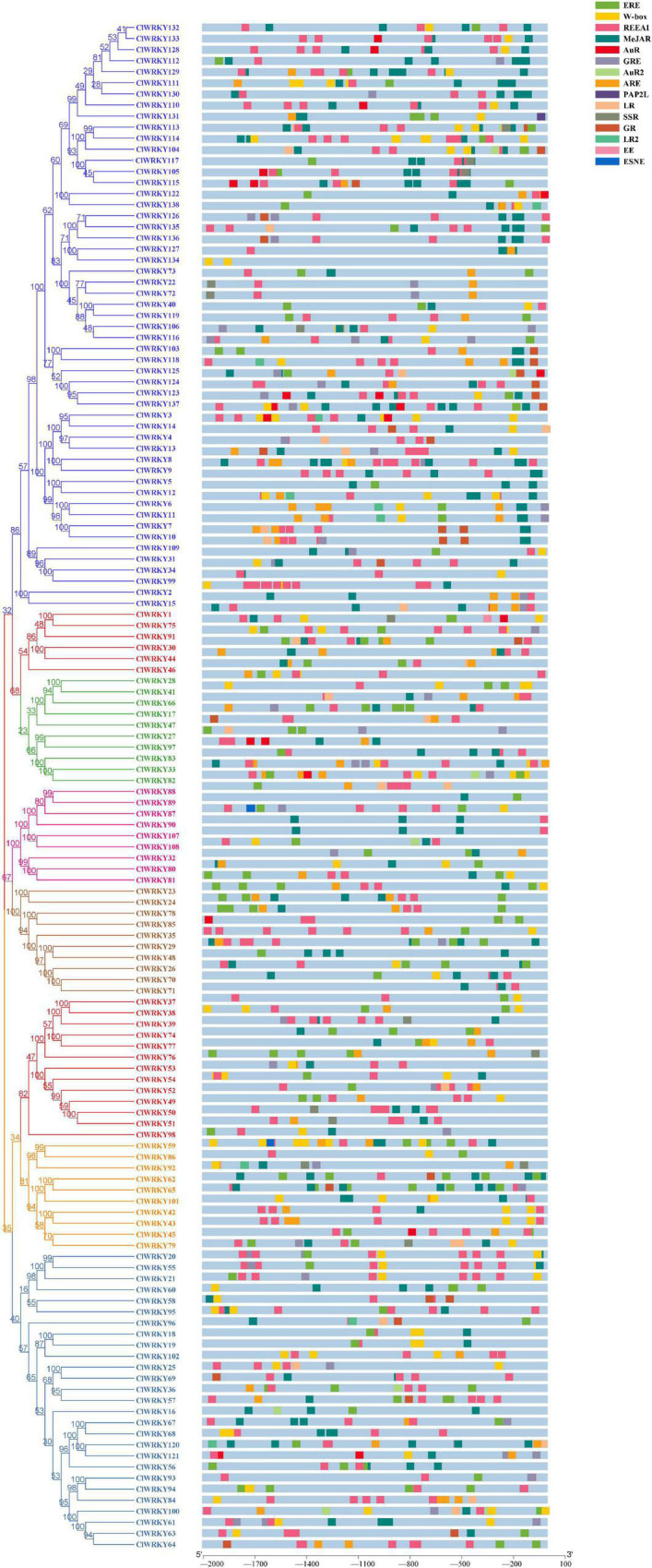
*Cis*-acting element analysis in the promoter region of the *ClWRKY* family. The PlantCARE software was used to search the *cis*-acting elements. Abbreviation of important *cis*-acting elements are as follows: EE, endosperm expression; ARE, auxin-responsive elements; GR, gibberellin responsiveness; LR, light responsiveness; REEAI, regulatory element essential for anaerobic induction; AuR, elements involved in auxin responsiveness; SSR, seed-specific regulation; MeJAR, methyl jasmonate responsiveness; ERE, ethylene-responsive elements; GRE, gibberellin responsive elements; ESNE, endosperm specific negative expression; LR2, elements involved in light response; PAP2L, plant-AP2 such as AuR2, part of auxin responsiveness; and W-box, recognition site of *WRKY* TFs.

Most of the genes have important *cis*-acting elements, such as auxin responsiveness (AuR) and gibberellin responsive elements (GRE), associated with flowering-related traits (Figure 6). W-box was most widely available among the *ClWRKY* family. *ClWRKY45* and *ClWRKY36*, the genes that resulted in high expression at the capitulum stage, had some important elements involved in endosperm expression, elements involved in auxin responsiveness, and ethylene-responsive elements (AuR, EE, and ERE), which are supposed to play a role in flower development. Methyl jasmonate (MeJAR), which plays important role in flowering induction, was present in most of the *ClWRKY* members, especially in group III members, which indicates that *WRKY* members may play a role in the overall development of a plant.

The *de novo* motif analysis in MEME identified 15 motifs (Supplementary Figure 2). Tomtom tool comparison showed homology with some important motifs, such as motifs similar to MEME12 and MEME14, which are involved in seed germination, pollen tube growth, and WRKY binding sites (Supplementary Table 5). The enriched motifs identified in MEME are mostly different from the motifs found in the PlantCare software. The simple enrichment analysis (SEA) in MEME identified 95 known enriched motifs. Among them, 49 motifs encoded ethylene, which is essential for plant growth and development. Other important motifs included are WRKY binding sites, TEOSINTE BRANCHED, cycloidea and PCF (TCP) binding sites, and so on (Supplementary Table 6).

### qRT-PCR Analyses

**FIGURE 7:**
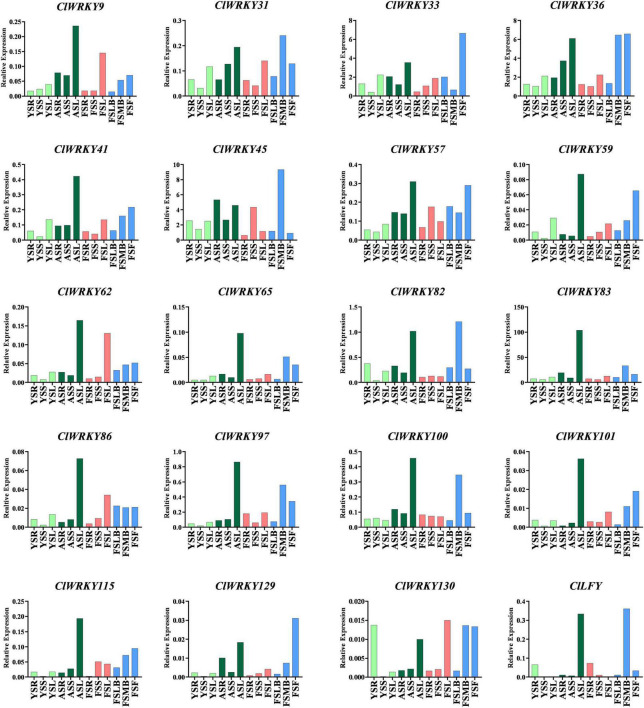
Expression analysis of the selected *ClWRKY* genes in different tissues (capitulum, leaf, stem, and root) at different stages (young, adult, and flowering). Expression of 19 genes: 10 genes were selected due to the presence of CpG island, nine genes were selected based on their close association with important *Arabidopsis* genes found in the phylogenetic tree built between *C. lavandulifolium* and *A. thaliana*. Abbreviations of different growth stages and tissues are as follows: YSR, young stage root; YSS, young stage stem; YSL, young stage leaf; ASR, adult stage root; ASS, adult stage stem; ASL, adult stage leaf; FSR, flowering stage root; FSS, flowering stage stem; FSL, flowering stage leaf; FSLB, Flowering stage little buds; FSMB, Flowering stage buds; FSF, Flowering stage first flower.

Different *CIWRKY* genes having special characters were selected on the basis of previous analysis. Total RNA was extracted from different tissues (root, stem, leaves, and flowers) *of C. lavandulifolium* at different stages (young, adult, and flowering). The expression pattern of 19 *WRKY* genes was observed in all tissues at different stages of the plant’s life span (Figure 7). Among them, 10 genes were selected due to the presence of CG-rich regions, and nine genes were selected as they showed close association with important *Arabidopsis WRKY* genes that are associated with floral traits. Most of the genes exhibited higher expression at the flowering stage in the capitulum tissues. The highest expression in the capitulum tissues at the flowering stage was exhibited by *ClWRKY36*, *ClWRKY45, ClWRKY57, ClWRKY59*, and *ClWRKY82* (Figure 7). The expression of these genes was higher in the flowering stage, especially in the first flowering (FF) stage and median bud stage, which suggests their role in flowering regulation. These genes were selected for qRT-PCR as they clustered with flowering-related At*WRKY* genes (Figure 2). On the basis of previous analysis, *ClWRKY36* clustered with *CIWRKY71*, so it might have a basic role in the control of flowering time and plant development, as *WRKY71* homolog was found to promote flowering in woodland strawberries (Lei et al., 2020). *ClWRKY31*, *ClWRKY33, ClWRKY41, ClWRKY97, ClWRKY100, ClWRKY129*, and *ClWRKY130*, which were selected on the basis of having CpG rich regions (Supplementary Table 1), showed higher expression in capitulum tissues (Figure 7). In addition, *ClWRKY31, ClWRKY100*, and *ClWRKY129* were detected with DNA methylation (Figure 5), which is linked with flowering regulation and is one of the basic sources of epigenetics. Interestingly, *ClWRKY83* remains stable across all the tissues and stages as it is constantly expressed in all samples of *C. lavandulifolium*. It might play a role in adaptability. Moreover, *ClWRKY83* showed close association with important *AtWRKY7* of *Arabidopsis*. The other *WRKY* member’s expressions showed less or non-significant changes at different stages in all tissues.

We also investigated *CILFY* (leafy), as it not only promotes flowering time but also influences other genes that regulate flowering-related traits (Yang et al., 2016). The expression of this gene was high in the median bud and adult leaf stages, but low in stem and root (Figure 7). Besides, *ClLFY* may promote flowering time in *C. lavandulifolium* by making association with *ClWRKY36 and ClWRKY45*.

## Discussion

*WRKY* TFs are one of the largest transcription families, playing an important role in plant development and resistance against different biotic and abiotic stresses. *WRKY* TFs are spread broadly across higher plants (Li et al., 2014). *WRKY* family investigation is limited to *C. morifolium* with 15 genes (Song A. et al., 2014). Being a model plant for chrysanthemums (Dong-ru et al., 2019), the characterization of the *WRKY* gene family in *C. lavandulifolium* is of great importance. For the first time, we identified 138 *WRKY* members in *C. lavandulifolium*. Out of 138 members, 118 were properly conserved in the “WRKYGQK” domain. Substitution of glutamine occurs instead of lysine in thirteen *WRKY* heptapeptide domains. This WRKYGKK sequence is reported to be a major variant in many studies (Song H. et al., 2014; Song et al., 2016a,b). *ClWRKY12* (WRKYGHK) “Q” replaced by “H” *ClWYKY20* and 21 (WRKYGQN) “K” replaced by “N,” *ClWRKY27* (WKKYGEQK) “K” replaced by “Q,” *ClWRKY*97 (WKKYGEK) “Q” replaced by “E,” and *ClWRKY131* (WRKNGQK) “Y” replaced by “N” as their respective domains were also reported in carrot (Nan and Gao, 2019). The variation in the heptapeptide domain of *WRKY* genes has been largely demonstrated (Yamasaki et al., 2005; Brand et al., 2013; Rinerson et al., 2015; Yang et al., 2020); however, variations in aa sequence were different from our findings. The variation in the WRKYGQK conserved domain mainly occurs from Q to K aa. The same kind of substitution was observed in the *WRKY* gene family of *Camelina sativa* (Song et al., 2020).

The *ClWRKY* gene family is composed of 138 genes, which is bigger than the *AtWRKY* gene family (Rushton et al., 2010). The *ClWRKY* family was classified into three groups. The classification of *ClWRKY* members was according to the classification of the *WRKY* family in *Arabidopsis*. Most of the *ClWRKY* members clustered in the corresponding groups. However, some of the *ClWRKY* members with one *WRKY* domain clustered in group I, which included, *ClWRKY58, 95, 60, 56, 20, 21*, and *ClWRKY55.* Among them, *ClWRKY20*, *21*, and *55* possessed a unique pattern “WNSIVV” which was conserved in these three members. Clustering of one domain *WRKY* of factors in group I is consistent with the study of the *WRKY* TF family in *Linum usitatissimum* (Yuan et al., 2021). The same results are reported in *Cicer arietinum* (Kumar et al., 2016). Some *WRKY* members with one conserved domain and clustered into group I have been reported in peanut (*Arachis hypogaea* L.) (Zhao et al., 2020).

The intron-exon structure seems to be diverse, starting from 1 to 9 introns. Diversification in intron-exon structure may play an important role in the evolution of genes (Xu et al., 2012). In this study, we have identified different intron constitutions within gene groups. Group I has four introns on average, group IIa has 5, group IIb has 4, and all other groups have an average of three introns. This suggests that the latter groups originated from group I and earlier subgroups of group II. The same trend of intron presence in different groups of *WRKY* members in eggplant was reported (Yang et al., 2020). A total of six motifs were detected; among them, two motifs were considered to be *WRKY* domains. The conserved domain “WRKYGQK” was almost included in all of the members; however, some of the *WRKY* members showed a slight variation as “WRKYGKK” domain as suggested by Liu et al. (2021) and Qu et al. (2021).

The *cis*-acting elements are involved in plant development and flowering induction. There are several elements, such as auxin, gibberellins, and jasmonic acid, which affect flowering-related traits. Jasmonates, cytokinins, and auxin control second bud growth in chrysanthemum (Sun et al., 2021). Auxin controls plant growth and development (Singla et al., 2006). The petal is the most important factor influencing the chrysanthemum’s ornamental value. Auxin signaling is associated with petal growth in chrysanthemums (Wang J. et al., 2017). AuR is found in most *ClWRKY* members and may play a role in plant development and flowering induction. GRE was abundant in *ClWRKY*, an important hormone associated with flowering induction in chrysanthemum (Dong et al., 2017). CmBBX24 regulates flowering in chrysanthemum in conjunction with gibberellin synthesis (Zhu et al., 2020). In addition, the key recognition site for the *WRKY* gene, the W-box containing the TGAC sequence, was observed in most *ClWRKY* members. The W-box contributes under abiotic stress conditions (Dhatterwal et al., 2019) by influencing the early senescence of rice flag leaves (Liu et al., 2016). The presence of flower-associated *cis*-acting elements in most genes indicates the potential of *ClWRKY* to play a role in flowering control in *C. lavandulifolium.*

DNA methylation has an impact on growth and development, with CG content, or CpG, being the most abundant source of DNA methylation (Dong-ru et al., 2019). Various methods such as methylation-sensitive amplification polymorphism and MSP have been applied to methylated genes (Herman et al., 1996; Coronel et al., 2018). In this study, we have identified ten genes with strong CpG content in the promoter region (Table 1), which may cause epigenetic changes in *C. lavandulifolium* (Chip and Dresselhaus, 2019). Three genes having methylated sites, i.e., *ClWRKY31, ClWRKY100*, and *ClWRKY129*, were identified on the basis of MSP results. These genes can regulate gene expression and may play a role in flowering-related traits, as they are expressed significantly in capitulum tissues at the flowering stage. *CmMET1* can affect methylation and regulate candidate genes associated with flowering in *C. morifolium* (Kang et al., 2021). DNA methylation changes in the promoter and gene body regions can influence gene expression and function of the protein (Miguel and Marum, 2011), the morphology of floral characters, photoperiod-related sensitivity, and fruit ripening (Sun et al., 2014; Xiao et al., 2020). We studied only four stages to detect methylation, and it will be better to explore the methylation status in more stages in the future.

This study focuses on the expression of *ClWRKY* members to find their role in plant development, especially their influence over flower induction. The expression analysis revealed variations of different genes in different organs. *ClWRKY45* showed significant expression at the flowering stage in the capitulum tissue, which may be related to some morphological characters and floral organ development. In the same fashion, *ClWRKY45* clustered with *Arabidopsis* At*WRKY71*, a prominent gene that controls flowering characteristics (Yu et al., 2016, 2018), providing strong evidence for our results. Moreover, *ClWRKY36* and *ClWRKY57* are significantly expressed in capitulum at the flowering stages and have close homology with *Arabidopsis AtWRKY2* and *AtWRKY34*, indicating the role of *CIWRKY36* and *ClWRKY57* in flowering, as *Arabidopsis* homologs are the key regulators of pollen development (Lei et al., 2017). *ClWRKY59* is expressed significantly in the first flower tissue and can positively regulate flowering, as *ClWRKY59* homolog, *AtWRKY75*, promoted flowering in *Arabidopsis* (Zhang et al., 2018). *ClWRKY82* exhibited higher expression in the median bud stage, which indicates its potential to play a role in flowering regulation. *AtWRKY7*, a *ClWRKY82* homolog, regulates plant growth (Chen et al., 2019). The qRT-PCR results indicate that *ClWRKY83* is a stable gene as it expresses across all the tissues at different stages of plant life and may play a role in adaptability. The higher expression of three genes in capitulum tissues, including *ClWRKY31, ClWRKY100*, and *ClWRKY129*, which are methylated as well, can play an important role in flowering-related traits. These results suggest that *ClWRKY33, ClWRKY36*, and *ClWRKY45*, along with *ClWRKY31*, *ClWRKY100*, and *ClWRKY129*, can play a crucial role in the genetic and epigenetic improvement of the chrysanthemum. *ClWRKY45, ClWRKY36*, and *ClWRK129*, in particular, have the potential to alter flowering-related traits. The overall investigation of the *CIWRKY* domain is providing theoretical insight into *C. lavandulifolium* transcriptional activation and inactivation mechanisms for the improvement of plant development.

## Conclusion

This study aimed to identify the *WRKY* gene family in *C. lavandulifolium* and explore the potential of *WRKY* genes for genetic improvement. A total of 138 *WRKY* genes were analyzed in *C. lavandulifolium*. All the data results are unique to explore the underlying mechanism of the WRKY TF in *C. lavandulifolium* plant improvement. The gene structure analysis suggested the vast variation among *WRKY* members. The *WRKY* domain remains conserved across all the members of this family. Ten *WRKY* genes possessed CG-rich content in the promoter region where three genes exhibit methylation status. This research can provide a basis to study the role of *WRKY* genes in the improvement of chrysanthemum plants, especially in flowering traits.

## Data Availability Statement

The datasets presented in this study can be found in online repositories. The names of the repository/repositories and accession number(s) can be found in the article/Supplementary Material.

## Author Contributions

WZ designed the research project, coordinated, and supervised the study. MA performed all the experiments and wrote the manuscript. MA and KD did most of the experimental work and analyzed the data. WuY, AP, and WaY performed specific experiments during the research. All authors read and approved the final manuscript.

## Conflict of Interest

The authors declare that the research was conducted in the absence of any commercial or financial relationships that could be construed as a potential conflict of interest.

## Publisher’s Note

All claims expressed in this article are solely those of the authors and do not necessarily represent those of their affiliated organizations, or those of the publisher, the editors and the reviewers. Any product that may be evaluated in this article, or claim that may be made by its manufacturer, is not guaranteed or endorsed by the publisher.

## Footnotes

1
http://gsds.gao-lab.org/

2
https://www.ncbi.nlm.nih.gov/Structure/bwrpsb/bwrpsb.cgi

3
https://meme-suite.org/meme/tools/meme

4
https://meme-suite.org/meme/tools/sea

5
thermofisher.com

6
http://gsds.cbi.pku.edu.cn/

## References

[B1] Bailey T. L. Elkan C. (1994). Fitting a mixture model by expectation maximization to discover motifs in biopolymers. *Proc. Int. Conf. Intell. Syst. Mol. Biol.* 2 28–36. 7584402

[B2] Bailey T. L. Grant C. E. (2021). SEA: Simple Enrichment Analysis of motifs. *bioRxiv* [Preprint]. 10.1101/2021.09.02.458722

[B3] Bailey T. L. Johnson J. Grant C. E. Noble W. S. (2015). The MEME Suite. *Nucleic Acids Res.* 43 W39–W49. 25953851 10.1093/nar/gkv416 PMC4489269

[B4] Bi C. Xu Y. Ye Q. Yin T. Ye N. (2016). Genome-wide identification and characterization of WRKY gene family in Salix suchowensis. *PeerJ* 4:e2437. 10.7717/peerj.2437 27651997 PMC5018666

[B5] Bi M. Li X. Yan X. Liu D. Gao G. Zhu P. (2021). Chrysanthemum WRKY15-1 promotes resistance to Puccinia horiana Henn. *via* the salicylic acid signaling pathway. *Hortic Res.* 8:6. 10.1038/s41438-020-00436-4 33384451 PMC7775453

[B6] Brand L. H. Fischer N. M. Harter K. Kohlbacher O. Wanke D. (2013). Elucidating the evolutionary conserved DNA-binding specificities of WRKY transcription factors by molecular dynamics and *in vitro* binding assays. *Nucleic Acids Res.* 41 9764–9778. 10.1093/nar/gkt732 23975197 PMC3834811

[B7] Cai Y. Chen X. Xie K. Xing Q. Wu Y. Li J. (2014). Dlf1, a WRKY transcription factor, is involved in the control of flowering time and plant height in rice. *PLoS One* 9:e102529. 10.1371/journal.pone.0102529 25036785 PMC4103817

[B8] Chen C. Chen H. Zhang Y. Thomas H. R. Frank M. H. He Y. (2020). TBtools: An Integrative Toolkit Developed for Interactive Analyses of Big Biological Data. *Mol. Plant* 13 1194–1202. 10.1016/j.molp.2020.06.009 32585190

[B9] Chen H. Lai Z. Shi J. Xiao Y. Chen Z. Xu X. (2010). Roles of arabidopsis WRKY18, WRKY40 and WRKY60 transcription factors in plant responses to abscisic acid and abiotic stress. *BMC Plant Biol.* 10:281. 10.1186/1471-2229-10-281 21167067 PMC3023790

[B10] Chen W. Hao W. J. Xu Y. X. Zheng C. Ni D. J. Yao M. Z. (2019). Isolation and characterization of CsWRKY7, a subgroup IId WRKY transcription factor from Camellia sinensis, linked to development in arabidopsis. *Int. J. Mol. Sci.* 20:2815. 10.3390/ijms20112815 31181825 PMC6600228

[B11] Chip I. Dresselhaus T. (2019). Chapter 12 and Reproductive Development in Cereals Using Chromatin. *Cereal Genomics* 2072 141–156. 10.1007/978-1-4939-9865-4_12 31541444

[B12] Coronel C. J. González A. I. Ruiz M. L. Polanco C. (2018). Analysis of somaclonal variation in transgenic and regenerated plants of Arabidopsis thaliana using methylation related metAFLP and TMD markers. *Plant Cell Rep.* 37 137–152. 10.1007/s00299-017-2217-x 29038910

[B13] Dhatterwal P. Basu S. Mehrotra S. Mehrotra R. (2019). Genome wide analysis of W-box element in Arabidopsis thaliana reveals TGAC motif with genes down regulated by heat and salinity. *Sci Rep.* 9:1681. 10.1038/s41598-019-38757-7 30737427 PMC6368537

[B14] Dong B. Deng Y. Wang H. Gao R. Stephen G. K. Chen S. (2017). Gibberellic acid signaling is required to induce flowering of chrysanthemums grown under both short and long days. *Int. J. Mol. Sci.* 18:1259. 10.3390/ijms18061259 28604637 PMC5486081

[B15] Dong-ru K. Si-lan D. Kang G. Fan Z. Hong L. (2019). Morphological variation of Chrysanthemum lavandulifolium induced by 5-azaC treatment. *Sci. Hortic.* 257:108645. 10.1016/j.scienta.2019.108645

[B16] Duan M. R. Nan J. Liang Y. H. Mao P. Lu L. Li L. (2007). DNA binding mechanism revealed by high resolution crystal structure of Arabidopsis thaliana WRKY1 protein. *Nucleic Acids Res.* 35 1145–1154. 10.1093/nar/gkm001 17264121 PMC1851648

[B17] Fan Q. Song A. Jiang J. Zhang T. Sun H. Wang Y. (2016). CmWRKY1 enhances the dehydration tolerance of chrysanthemum through the regulation of ABA-aociated genes. *PLoS One* 11:e0150572. 10.1371/journal.pone.0150572 26938878 PMC4777562

[B18] Fan Q. Song A. Xin J. Chen S. Jiang J. Wang Y. (2015). CmWRKY15 facilitates Alternaria tenuissima infection of chrysanthemum. *PLoS One* 10:e0143349. 10.1371/journal.pone.0143349 26600125 PMC4658048

[B19] Fu J. Wang Y. Huang H. Zhang C. Dai S. (2013). Reference gene selection for RT-qPCR analysis of Chrysanthemum lavandulifolium during its flowering stages. *Mol. Breed.* 31 205–215. 10.1007/s11032-012-9784-x

[B20] Gupta S. Stamatoyannopoulos J. A. Bailey T. L. Noble W. S. (2007). Quantifying similarity between motifs. *Genome Biol.* 8:R24, 10.1186/gb-2007-8-2-r24 17324271 PMC1852410

[B21] He L. Wu Y. H. Zhao Q. Wang B. Liu Q. L. Zhang L. (2018). Chrysanthemum DgWRKY2 gene enhances tolerance to salt stress in transgenic chrysanthemum. *Int. J. Mol. Sci.* 19:2062. 10.3390/ijms19072062 30012947 PMC6073511

[B22] Herman J. G. Graff J. R. Myohanen S. Nelkin B. D. Baylin S. B. (1996). Methylation-specific PCR: A novel PCR assay for methylation status of CpG islands (DNA methylation/tumor suppressor genes/pl6/p15). *Med. Sci.* 93 9821–9826. 10.1073/pnas.93.18.9821 8790415 PMC38513

[B23] Hu B. Jin J. Guo A. Y. Zhang H. Luo J. Gao G. (2015). GSDS 2.0: An upgraded gene feature visualization server. *Bioinformatics* 31 1296–1297. 10.1093/bioinformatics/btu817 25504850 PMC4393523

[B24] Huang H. Cao H. Niu Y. Dai S. (2012). Expression Analysis of Nudix Hydrolase Genes in Chrysanthemum lavandulifolium. *Plant Mol. Biol. Rep.* 30 973–982. 10.1007/s11105-011-0401-7

[B25] Ishiguro S. Nakamura K. (1994). Characterization of a cDNA encoding a novel DNA-binding protein, SPF1, that recognizes SP8 sequences in the 5’ upstream regions of genes coding for sporamin and β-amylase from sweet potato. *MGG Mol. Gen. Genet.* 244 563–571. 10.1007/BF00282746 7969025

[B26] Jaffar M. A. Song A. Faheem M. Chen S. Jiang J. Liu C. (2016). Involvement of CmWRKY10 in drought tolerance of chrysanthemum through the ABA-signaling pathway. *Int. J. Mol. Sci.* 17:693. 10.3390/ijms17050693 27187353 PMC4881519

[B27] Kang D. R. Zhu Y. Li S. L. Ai P. H. Khan M. A. Ding H. X. (2021). Transcriptome analysis of differentially expressed genes in chrysanthemum MET1 RNA interference lines. *Physiol. Mol. Biol. Plants* 27 1455–1468. 10.1007/s12298-021-01022-1 34366589 PMC8295425

[B28] Kumar K. Srivastava V. Purayannur S. Kaladhar V. C. Cheruvu P. J. Verma P. K. (2016). WRKY domain-encoding genes of a crop legume chickpea (Cicer arietinum): Comparative analysis with Medicago truncatula WRKY family and characterization of group-III gene(s). *DNA Res.* 23 225–239. 10.1093/dnares/dsw010 27060167 PMC4909309

[B29] Kumar S. Stecher G. Li M. Knyaz C. Tamura K. (2018). MEGA X: Molecular evolutionary genetics analysis across computing platforms. *Mol. Biol. Evol.* 35 1547–1549. 10.1093/molbev/msy096 29722887 PMC5967553

[B30] Lei R. Li X. Ma Z. Lv Y. Hu Y. Yu D. (2017). Arabidopsis WRKY2 and WRKY34 transcription factors interact with VQ20 protein to modulate pollen development and function. *Plant J.* 91 962–976. 10.1111/tpj.13619 28635025

[B31] Lei Y. Sun Y. Wang B. Yu S. Dai H. Li H. (2020). Woodland strawberry WRKY71 acts as a promoter of flowering *via* a transcriptional regulatory cascade. *Hortic Res.* 7:137. 10.1038/s41438-020-00355-4 32922809 PMC7458929

[B32] Lescot M. Déhais P. Thijs G. Marchal K. Moreau Y. Van De Peer Y. (2002). PlantCARE, a database of plant cis-acting regulatory elements and a portal to tools for in silico analysis of promoter sequences. *Nucleic Acids Res.* 30 325–327. 10.1093/nar/30.1.325 11752327 PMC99092

[B33] Li H. L. Guo D. Yang Z. P. Tang X. Peng S. Q. (2014). Genome-wide identification and characterization of WRKY gene family in Hevea brasiliensis. *Genomics* 104 14–23. 10.1016/j.ygeno.2014.04.004 24793160

[B34] Li P. Song A. Gao C. Jiang J. Chen S. Fang W. (2015a). The over-expression of a chrysanthemum WRKY transcription factor enhances aphid resistance. *Plant Physiol. Biochem.* 95 26–34. 10.1016/j.plaphy.2015.07.002 26184088

[B35] Li P. Song A. Gao C. Wang L. Wang Y. Sun J. (2015b). Chrysanthemum WRKY gene CmWRKY17 negatively regulates salt stress tolerance in transgenic chrysanthemum and Arabidopsis plants. *Plant Cell Rep.* 34 1365–1378. 10.1007/s00299-015-1793-x 25893877

[B36] Li S. Fu Q. Chen L. Huang W. Yu D. (2011). Arabidopsis thaliana WRKY25, WRKY26, and WRKY33 coordinate induction of plant thermotolerance. *Planta* 233 1237–1252. 10.1007/s00425-011-1375-2 21336597

[B37] Li S. Fu Q. Huang W. Yu D. (2009). Functional analysis of an Arabidopsis transcription factor WRKY25 in heat stress. *Plant Cell Rep.* 28 683–693. 10.1007/s00299-008-0666-y 19125253

[B38] Li W. Wang H. Yu D. (2016). Arabidopsis WRKY Transcription Factors WRKY12 and WRKY13 Oppositely Regulate Flowering under Short-Day Conditions. *Mol. Plant* 9 1492–1503. 10.1016/j.molp.2016.08.003 27592586

[B39] Liang Q. Y. Wu Y. H. Wang K. Bai Z. Y. Liu Q. L. Pan Y. Z. (2017). Chrysanthemum WRKY gene DgWRKY5 enhances tolerance to salt stress in transgenic chrysanthemum. *Sci. Rep.* 7:4799. 10.1038/s41598-017-05170-x 28684847 PMC5500475

[B40] Lin L. Z. Harnly J. M. (2010). Identification of the phenolic components of chrysanthemum flower (Chrysanthemum morifolium Ramat). *Food Chem.* 120 319–326. 10.1016/j.foodchem.2009.09.083

[B41] Liu J. Wang X. Chen Y. Liu Y. Wu Y. Ren S. (2021). Identification, evolution and expression analysis of WRKY gene family in Eucommia ulmoides. *Genomics* 113 3294–3309. 10.1016/j.ygeno.2021.05.011 34022347

[B42] Liu L. Xu W. Hu X. Liu H. Lin Y. (2016). W-box and G-box elements play important roles in early senescence of rice flag leaf. *Sci. Rep.* 6:20881. 10.1038/srep20881 26864250 PMC4749992

[B43] Liu Q. L. Xu K. D. Pan Y. Z. Jiang B. B. Liu G. L. Jia Y. (2014). Functional Analysis of a Novel Chrysanthemum WRKY Transcription Factor Gene Involved in Salt Tolerance. *Plant Mol. Biol. Rep.* 32 282–289. 10.1007/s11105-013-0639-3

[B44] Liu Q. L. Zhong M. Li S. Pan Y. Z. Jiang B. B. Jia Y. (2013). Overexpression of a chrysanthemum transcription factor gene, DgWRKY3, intobacco enhances tolerance to salt stress. *Plant Physiol. Biochem.* 69 27–33. 10.1016/j.plaphy.2013.04.016 23707882

[B45] Livak K. J. Schmittgen T. D. (2001). Analysis of relative gene expression data using real-time quantitative PCR and the 2-ΔΔCT method. *Methods* 25 402–408. 10.1006/meth.2001.1262 11846609

[B46] Luo X. Sun X. Liu B. Zhu D. Bai X. Cai H. (2013). Ectopic Expression of a WRKY Homolog from Glycine soja Alters Flowering Time in Arabidopsis. *PLoS One* 8:e73295. 10.1371/journal.pone.0073295 23991184 PMC3753250

[B47] Ma Z. Li W. Wang H. Yu D. (2020). WRKY transcription factors WRKY12 and WRKY13 interact with SPL10 to modulate age-mediated flowering. *J. Integr. Plant Biol.* 62 1659–1673. 10.1111/jipb.12946 32396254

[B48] Miguel C. Marum L. (2011). An epigenetic view of plant cells cultured *in vitro*: Somaclonal variation and beyond. *J. Exp. Bot.* 62 3713–3725. 10.1093/jxb/err155 21617249

[B49] Nan H. Gao L. Z. (2019). Genome-wide analysis of WRKY genes and their response to hormone and mechanic stresses in carrot. *Front. Genet.* 10:363. 10.3389/fgene.2019.00363 31191596 PMC6504813

[B50] Nguyen T. K. Lim J. H. (2019). Tools for Chrysanthemum genetic research and breeding: Is genotyping-by-sequencing (GBS) the best approach? *Hortic. Environ. Biotechnol.* 60 625–635. 10.1007/s13580-019-00160-6

[B51] Phukan U. J. Jeena G. S. Shukla R. K. (2016). WRKY transcription factors: Molecular regulation and stress responses in plants. *Front. Plant Sci.* 7:760. 10.3389/fpls.2016.00760 27375634 PMC4891567

[B52] Qu R. Cao Y. Tang X. Sun L. Wei L. Wang K. (2021). Identification and expression analysis of the WRKY gene family in Isatis indigotica. *Gene* 783:145561. 10.1016/j.gene.2021.145561 33705810

[B53] Ramos R. N. Martin G. B. Pombo M. A. Rosli H. G. (2021). WRKY22 and WRKY25 transcription factors are positive regulators of defense responses in Nicotiana benthamiana. *Plant Mol Biol.* 105 65–82. 10.1007/s11103-020-01069-w 32909182

[B54] Rinerson C. I. Rabara R. C. Tripathi P. Shen Q. J. Rushton P. J. (2015). The evolution of WRKY transcription factors. *BMC Plant Biol.* 15:66. 10.1186/s12870-015-0456-y 25849216 PMC4350883

[B55] Rombauts S. Déhais P. Van Montagu M. Rouzé P. (1999). PlantCARE, a plant cis-acting regulatory element database. *Nucleic Acids Res.* 27 295–296. 10.1093/nar/27.1.295 9847207 PMC148162

[B56] Rushton P. J. Somssich I. E. Ringler P. Shen Q. J. (2010). WRKY transcription factors. *Trends Plant Sci.* 15 247–258. 10.1016/j.tplants.2010.02.006 20304701

[B57] Scarpeci T. E. Zanor M. I. Mueller-Roeber B. Valle E. M. (2013). Overexpression of AtWRKY30 enhances abiotic stress tolerance during early growth stages in Arabidopsis thaliana. *Plant Mol Biol.* 83 265–277. 10.1007/s11103-013-0090-8 23794142

[B58] Shinoyama H. Aida R. Ichikawa H. Nomura Y. Mochizuki A. (2012). Genetic engineering of chrysanthemum (Chrysanthemum morifolium): Current progress and perspectives. *Plant Biotechnol.* 29 323–337. 10.5511/plantbiotechnology.12.0521a

[B59] Singla B. Chugh A. Khurana J. P. Khurana P. (2006). An early auxin-responsive Aux/IAA gene from wheat (Triticum aestivum) is induced by epibrassinolide and differentially regulated by light and calcium. *J. Exp. Bot.* 57 4059–4070. 10.1093/jxb/erl182 17077182

[B60] Soares J. M. Weber K. C. Qiu W. Stanton D. Mahmoud L. M. Wu H. (2020). The vascular targeted citrus FLOWERING LOCUS T3 gene promotes non-inductive early flowering in transgenic Carrizo rootstocks and grafted juvenile scions. *Sci. Rep.* 10:21404. 10.1038/s41598-020-78417-9 33293614 PMC7722890

[B61] Song A. Li P. Jiang J. Chen S. Li H. Zeng J. (2014). Phylogenetic and transcription analysis of chrysanthemum WRKY transcription factors. *Int. J. Mol. Sci.* 15 14442–14455. 10.3390/ijms150814442 25196345 PMC4159861

[B62] Song H. Wang P. Hou L. Zhao S. Zhao C. Xia H. (2016a). Global analysis of WRKY genes and their response to dehydration and salt stress in soybean. *Front. Plant Sci.* 7:9. 10.3389/fpls.2016.00009 26870047 PMC4740950

[B63] Song H. Wang P. Lin J. Y. Zhao C. Bi Y. Wang X. (2016b). Genome-wide identification and characterization of WRKY gene family in peanut. *Front. Plant Sci.* 7:534. 10.3389/fpls.2016.00534 27200012 PMC4845656

[B64] Song H. Wang P. Nan Z. Wang X. (2014). The WRKY transcription factor genes in lotus japonicus. *Int. J. Genom.* 2014:420128. 10.1155/2014/420128 24745006 PMC3976811

[B65] Song Y. Cui H. Shi Y. Xue J. Ji C. Zhang C. (2020). Genome-wide identification and functional characterization of the Camelina sativa WRKY gene family in response to abiotic stress. *BMC Genom.* 21:786. 10.1186/s12864-020-07189-3 33176698 PMC7659147

[B66] Sun D. Zhang L. Yu Q. Zhang J. Li P. Zhang Y. (2021). Integrated Signals of Jasmonates, Sugars, Cytokinins and Auxin Influence the Initial Growth of the Second Buds of Chrysanthemum after Decapitation. *Biology* 10:440 10.3390/biology10050440 34065759 PMC8156878

[B67] Sun H. Guo Z. Gao L. Zhao G. Zhang W. Zhou R. (2014). DNA methylation pattern of Photoperiod-B1 is associated with photoperiod insensitivity in wheat (Triticum aestivum). *New Phytol.* 204 682–692. 10.1111/nph.12948 25078249

[B68] Turck F. Zhou A. Somssich I. E. (2004). Stimulus-dependent, promoter-specific binding of transcription factor WRKY1 to its native promoter and the defense-related gene PcPR1-1 in parsley. *Plant Cell* 16 2573–2585. 10.1105/tpc.104.024810 15367720 PMC520956

[B69] Wang J. Wang H. Ding L. Song A. Shen F. Jiang J. (2017). Transcriptomic and hormone analyses reveal mechanisms underlying petal elongation in Chrysanthemum morifolium ‘Jinba.’ *Plant Mol. Biol.* 93 593–606. 10.1007/s11103-017-0584-x 28108965

[B70] Wang K. Wu Y. H. Tian X. Q. Bai Z. Y. Liang Q. Y. Liu Q. L. (2017). Overexpression of DgWRKY4 enhances salt tolerance in chrysanthemum seedlings. *Front. Plant Sci.* 8:1592. 10.3389/fpls.2017.01592 28959270 PMC5604078

[B71] Wang Y. Li Y. He S. P. Gao Y. Wang N. N. Lu R. (2019). A cotton (Gossypium hirsutum) WRKY transcription factor (GhWRKY22) participates in regulating anther/pollen development. *Plant Physiol. Biochem.* 141 231–239. 10.1016/j.plaphy.2019.06.005 31195253

[B72] Warmerdam S. Sterken M. G. Sukarta O. C. A. Van Schaik C. C. Oortwijn M. E. P. Lozano-Torres J. L. (2020). The TIR-NB-LRR pair DSC1 and WRKY19 contributes to basal immunity of Arabidopsis to the root-knot nematode Meloidogyne incognita. *BMC Plant Biol.* 20:73. 10.1186/s12870-020-2285-x 32054439 PMC7020509

[B73] Wen X. Li J. Wang L. Lu C. Gao Q. Xu P. (2022). The chrysanthemum lavandulifolium genome and the molecular mechanism underlying diverse capitulum types. *Hortic Res.* 9:uhab022. 10.1093/hr/uhab022 35039834 PMC8771455

[B74] Wu J. Zhang Y. Zhang H. Huang H. Folta K. M. Lu J. (2010). Whole genome wide expression profiles of Vitis amurensis grape responding to downy mildew by using Solexa sequencing technology. *BMC Plant Biol.* 10:234. 10.1186/1471-2229-10-234 21029438 PMC3017854

[B75] Xiao K. Chen J. He Q. Wang Y. Shen H. Sun L. (2020). DNA methylation is involved in the regulation of pepper fruit ripening and interacts with phytohormones. *J. Exp. Bot.* 71 1928–1942. 10.1093/jxb/eraa003 31907544 PMC7242076

[B76] Xu G. Guo C. Shan H. Kong H. (2012). Divergence of duplicate genes in exon-intron structure. *Proc. Natl. Acad. Sci. U.S.A.* 109 1187–1192. 10.1073/pnas.1109047109 22232673 PMC3268293

[B77] Yamasaki K. Kigawa T. Inoue M. Tateno M. Yamasaki T. Yabuki T. (2005). Solution structure of an Arabidopsis WRKY DNA binding domain. *Plant Cell* 17 944–956. 10.1105/tpc.104.026435 15705956 PMC1069710

[B78] Yang C. Ye Y. Song C. Chen D. Jiang B. Wang Y. (2016). Cloning and functional identification of the AcLFY gene in Allium cepa. *Biochem. Biophys. Res. Commun.* 473 1100–1105. 10.1016/j.bbrc.2016.04.022 27074580

[B79] Yang L. Fu J. Qi S. Hong Y. Huang H. Dai S. (2017). Molecular cloning and function analysis of ClCRY1a and ClCRY1b, two genes in Chrysanthemum lavandulifolium that play vital roles in promoting floral transition. *Gene* 617 32–43. 10.1016/j.gene.2017.02.020 28216039

[B80] Yang Y. Liu J. Zhou X. Liu S. Zhuang Y. (2020). Identification of WRKY gene family and characterization of cold stress-responsive WRKY genes in eggplant. *PeerJ* 8:e8777. 10.7717/peerj.8777 32211240 PMC7083166

[B81] Yokotani N. Shikata M. Ichikawa H. Mitsuda N. Ohme-Takagi M. Minami E. (2018). OsWRKY24, a blast-disease responsive transcription factor, positively regulates rice disease resistance. *J. Gen. Plant Pathol.* 84 85–91. 10.1007/s10327-018-0768-5

[B82] Yu Y. Liu Z. Wang L. Kim S. G. Seo P. J. Qiao M. (2016). WRKY71 accelerates flowering *via* the direct activation of FLOWERING LOCUS T and LEAFY in Arabidopsis thaliana. *Plant J.* 85 96–106. 10.1111/tpj.13092 26643131

[B83] Yu Y. Wang L. Chen J. Liu Z. Park C. M. Xiang F. (2018). WRKY71 Acts Antagonistically Against Salt-Delayed Flowering in Arabidopsis thaliana. *Plant Cell Physiol.* 59 414–422. 10.1093/pcp/pcx201 29272465

[B84] Yuan H. Guo W. Zhao L. Yu Y. Chen S. Tao L. (2021). Genome-wide identification and expression analysis of the WRKY transcription factor family in flax (Linum usitatissimum L.). *BMC Genom.* 22:375. 10.1186/s12864-021-07697-w 34022792 PMC8141250

[B85] Yue J. Zhu C. Zhou Y. Niu X. Miao M. Tang X. (2018). Transcriptome analysis of differentially expressed unigenes involved in flavonoid biosynthesis during flower development of Chrysanthemum morifolium ‘Chuju.’ *Sci. Rep.* 8:13414. 10.1038/s41598-018-31831-6 30194355 PMC6128863

[B86] Zhang L. Chen L. Yu D. (2018). Transcription factor WRKY75 interacts with DELLA proteins to affect flowering. *Plant Physiol.* 176 790–803. 10.1104/pp.17.00657 29133369 PMC5761768

[B87] Zhang W. Gao T. Li P. Tian C. Song A. Jiang J. (2020). Chrysanthemum CmWRKY53 negatively regulates the resistance of chrysanthemum to the aphid Macrosiphoniella sanborni. *Hortic Res.* 7:109, 10.1038/s41438-020-0334-0 32637137 PMC7327015

[B88] Zhao N. He M. Li L. Cui S. Hou M. Wang L. (2020). Identification and expression analysis of WRKY gene family under drought stress in peanut (Arachis hypogaea L.). *PLoS One* 15:e0231396. 10.1371/journal.pone.0231396 32271855 PMC7144997

[B89] Zhou Q. Y. Tian A. G. Zou H. F. Xie Z. M. Lei G. Huang J. (2008). Soybean WRKY-type transcription factor genes, GmWRKY13, GmWRKY21, and GmWRKY54, confer differential tolerance to abiotic stresses in transgenic Arabidopsis plants. *Plant Biotechnol. J.* 6 486–503. 10.1111/j.1467-7652.2008.00336.x 18384508

[B90] Zhou X. Jiang Y. Yu D. (2011). WRKY22 transcription factor mediates dark-induced leaf senescence in Arabidopsis. *Mol. Cells* 31 303–313. 10.1007/s10059-011-0047-1 21359674 PMC3933965

[B91] Zhu L. Guan Y. Liu Y. Zhang Z. Jaffar M. A. Song A. (2020). Regulation of flowering time in chrysanthemum by the R2R3 MYB transcription factor CmMYB2 is associated with changes in gibberellin metabolism. *Hortic Res.* 7:96. 10.1038/s41438-020-0317-1 32637124 PMC7326907

