# Supplementary figures and images for: Characterization of WRKY Gene Family in Whole-Genome and Exploration of Flowering Improvement Genes in Chrysanthemum lavandulifolium
Source: Front Plant Sci. 2022 Apr 26;13:861193. doi: 10.3389/fpls.2022.861193 (PMC9087852; doi:10.3389/fpls.2022.861193)

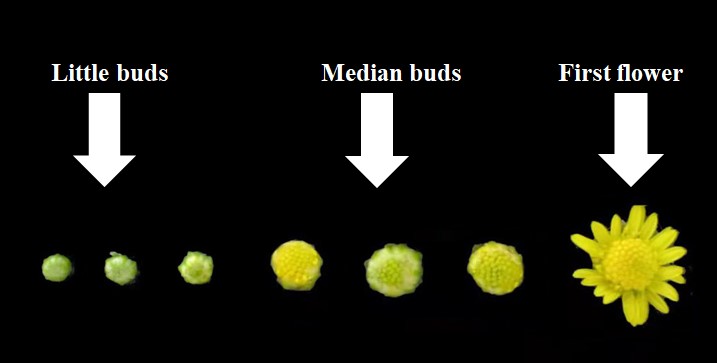

Supplement: Supplementary file 7 [file Image_1.JPEG]

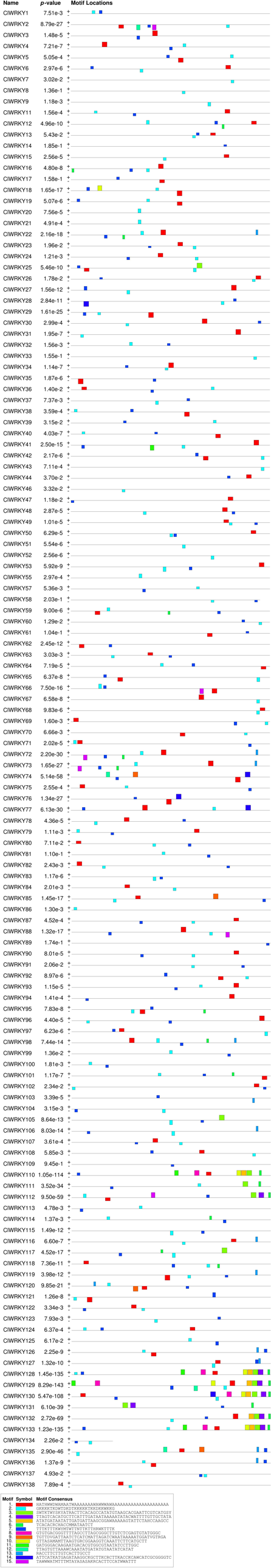

Supplement: Supplementary file 8 [file Image_2.JPEG]
